# An extended focused assessment with sonography in trauma ultrasound tissue-mimicking phantom for developing automated diagnostic technologies

**DOI:** 10.3389/fbioe.2023.1244616

**Published:** 2023-11-14

**Authors:** Sofia I. Hernandez-Torres, Carlos Bedolla, David Berard, Eric J. Snider

**Affiliations:** Organ Support and Automation Technologies Group, U.S. Army Institute of Surgical Research, JBSA Fort Sam Houston, San Antonio, TX, United States

**Keywords:** ultrasound imaging, tissue phantom, artificial intelligence, image classification, triage, abdominal hemorrhage, pneumothorax, hemothorax

## Abstract

**Introduction:** Medical imaging-based triage is critical for ensuring medical treatment is timely and prioritized. However, without proper image collection and interpretation, triage decisions can be hard to make. While automation approaches can enhance these triage applications, tissue phantoms must be developed to train and mature these novel technologies. Here, we have developed a tissue phantom modeling the ultrasound views imaged during the enhanced focused assessment with sonography in trauma exam (eFAST).

**Methods:** The tissue phantom utilized synthetic clear ballistic gel with carveouts in the abdomen and rib cage corresponding to the various eFAST scan points. Various approaches were taken to simulate proper physiology without injuries present or to mimic pneumothorax, hemothorax, or abdominal hemorrhage at multiple locations in the torso. Multiple ultrasound imaging systems were used to acquire ultrasound scans with or without injury present and were used to train deep learning image classification predictive models.

**Results:** Performance of the artificial intelligent (AI) models trained in this study achieved over 97% accuracy for each eFAST scan site. We used a previously trained AI model for pneumothorax which achieved 74% accuracy in blind predictions for images collected with the novel eFAST tissue phantom. Grad-CAM heat map overlays for the predictions identified that the AI models were tracking the area of interest for each scan point in the tissue phantom.

**Discussion:** Overall, the eFAST tissue phantom ultrasound scans resembled human images and were successful in training AI models. Tissue phantoms are critical first steps in troubleshooting and developing medical imaging automation technologies for this application that can accelerate the widespread use of ultrasound imaging for emergency triage.

## 1 Introduction

**FIGURE 1:**
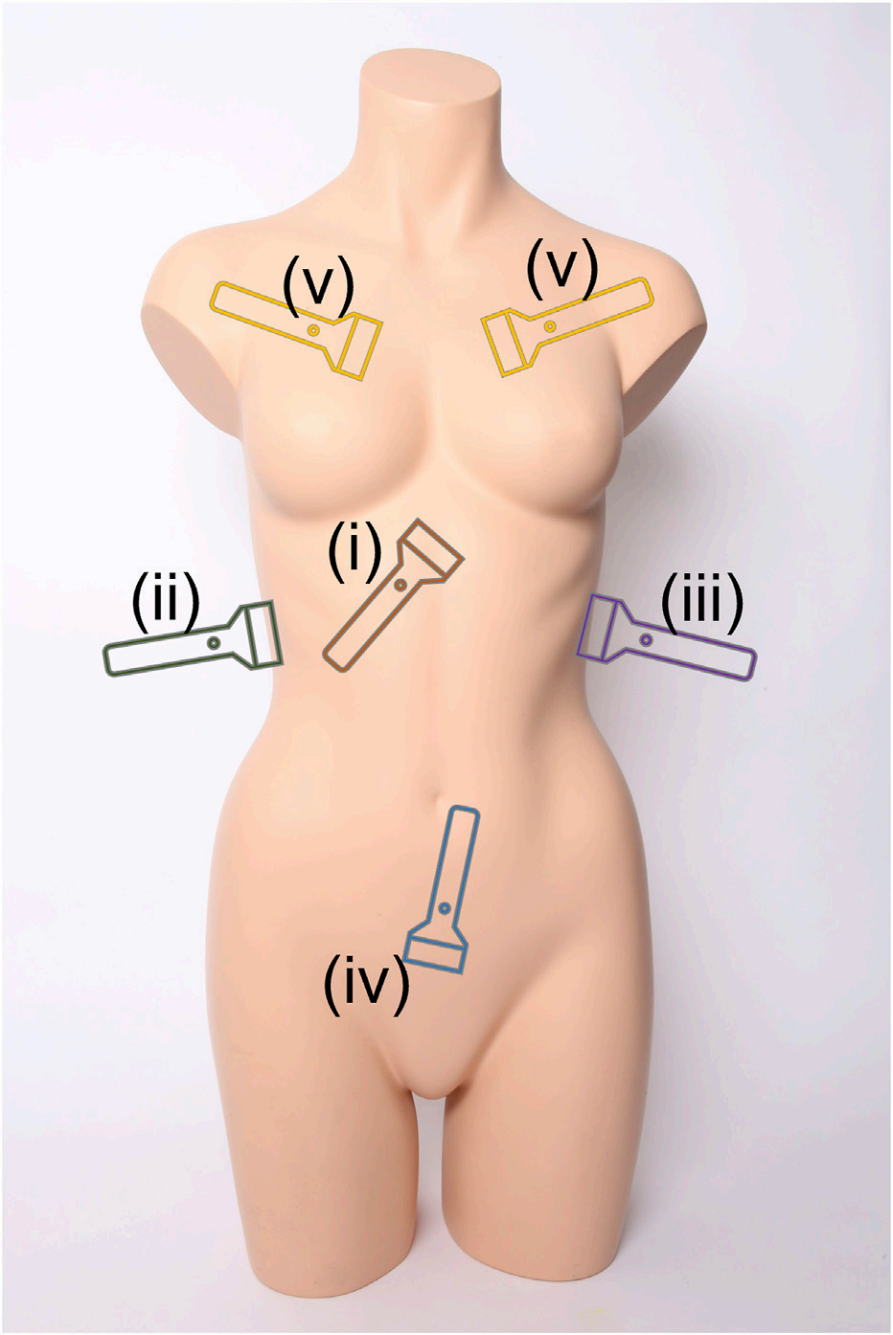
Ultrasound scan points for the extended Focused Assessment with Sonography for Trauma (eFAST) exam. Views include (i) subxiphoid, (ii) right upper quadrant, (iii) left upper quadrant, (iv) pelvic, and (v) intercostal scan points. Image obtained from creative commons as: “Torso” by Tim Reckmann | a59.de is licensed under CC BY 2.0 DEED and was modified to include eFAST scan location markers.

Medical triage is essential for prioritizing limited resources to ensure that medical intervention can be provided to the most urgent cases during emergency medicine. This extends to military medicine where triage is critical for prioritizing casualty evacuation (Ball and Keenan, 2015; Townsend and Lasher, 2018). A range of imaging-based and differential diagnosis approaches exist for patient triage depending on the situation and resource availability. One widely used methodology is the Focused Assessment with Sonography for Trauma (FAST) exam to identify free intraperitoneal fluid in the abdomen or pericardial fluid using ultrasound (US) (Scalea et al., 1999; Bloom and Gibbons, 2021). This exam has been adopted by emergency departments across the world as a standard means of rapidly identifying internal hemorrhage so that timely surgical intervention can be administered (Rozycki et al., 1995). The FAST exam is comprised of four scan points to assess for free fluid as diagrammed in Figure 1 (Bloom and Gibbons, 2021):

i. Subxiphoid view for evaluating the pericardial space for free fluid.
ii. Right Upper Quadrant (RUQ) view for assessing the hepatorenal recess or Morrison’s pouch, liver, and lower right thorax for abdominal hemorrhage (AH) and hemothorax (HTX).
iii. Left Upper Quadrant (LUQ) view evaluates the splenorenal recess, spleen, and lower left thorax for AH and HTX.
iv. Pelvic view scans the rectovesical or rectouterine pouch for AH.

The FAST protocol is often extended (eFAST) to include thoracic evaluation for identifying air in the pleural cavity—known as pneumothorax (PTX) (Kirkpatrick et al., 2004; Maximus et al., 2018). Untreated PTX can lead to a tension PTX which can impact cardiac venous return and result in shock (Jalota Sahota and Sayad, 2023). The eFAST protocol adds the following set of scan points diagrammed in Figure 1:

v. Multiple Intercostal space views on the left and right chest to identify lung sliding or the absence of motion due to air in the pleural cavity (Husain et al., 2012)

As the eFAST protocol is critical for triaging a number of potential conditions following trauma, it is essential that images are collected and interpreted correctly so that a proper triage assessment can be provided. As a result, image acquisition and interpretation require extensive training. While often present in emergency medical centers, in rural or remote medicine and combat casualty care, when resources are stretched thin, expertise for conducting an eFAST exam may not exist (Harper and Myers, 2008).

In response, the eFAST protocol in part or in its entirety can be automated to make this triage methodology more widely available and consistent. Image acquisition automation may take the form of augmented reality overlays to guide a user to proper scan points (Farshad-Amacker et al., 2020), imaging feedback to direct the user to proper scan points (Brattain et al., 2021), or utilize computer vision guided robotics to estimate body pose (Esteva et al., 2021; Zheng et al., 2022) and capture proper images fully autonomously. Image interpretation automation can utilize state-of-the-art deep learning artificial intelligent (AI) networks to create predictive models (Krizhevsky et al., 2012; Boice et al., 2022b) that categorically classify US images, provide masks (Hatamizadeh et al., 2022) or bounding box overlays (Redmon and Farhadi, 2018) of regions of interest in captured images, and even integration with ultrasound equipment for real-time deployment for rapid triage decision making. All of these automation methods will require large data sets to properly train and calibrate for this triage application and, thus, require training platforms for image acquisition and testing automation implementation.

Due to anatomical differences, this is challenging to accomplish with animal models and not feasible to do in clinical research due to the number of images or iterative tests needed to tune these automation approaches. Instead, ultrasound tissue phantom trainers are a logical starting point. Currently, there are some commercially available FAST or eFAST training models that have varying levels of real-time imaging capabilities. The SonoSkin trainer (Simulab, Seattle, WA) can simulate ultrasound scanning during an eFAST exam using a skin-like cover that can be placed over a mannequin or person. This in turn can be scanned with a simulated ultrasound probe to produce images at each scan site. The trainer includes a software system that shows predetermined ultrasound images when the probe scans specific anatomical locations in the skin cover (SonoSkin Ultrasound Diagnostic Wearable for FAST and eFAST Training, n.d.). However, these images are preloaded in the software and do not require the probe to be placed at the proper orientation relative to the scan point severely limiting its usability for training image acquisition and interpretation technologies.

Other more interactive trainers allow for real-time ultrasound scanning. Imaging Solutions (Brisbane, Australia) has a variety of FAST pediatric and adult abdomen phantoms. These phantoms have tissue and organs made of urethane-based resin and bone made of epoxy-based resin. These models allow for active ultrasound scanning of hemorrhage at imaging landmarks (Ultrasound Abdominal Examination Training Model ABDFAN, 2023). Similar to Imaging Solutions, CAE Healthcare (Sarasota, FL) offers a Blue Phantom FAST exam trainer. This training model is made of materials that mimic the ultrasound imaging characteristics of human tissue. The model has fluid spaces around the heart, spleen, bladder, and liver. Fluid can be inserted into these spaces by using a tube that runs through the leg to simulate a hemorrhage inside the abdomen (CAE Healthcare, 2022). All these models have effective mechanisms to simulate abdominal hemorrhage but lack the ability to simulate pneumothorax and hemothorax.

Apart from commercial trainers, some studies have focused on developing FAST scan ultrasound models. A previous research study (Al-Zogbi et al., 2021) developed a 3D-printed ultrasound model for FAST scanning. The bulk of the tissue in the phantom was molded with a combination gel, 70% Clear Ballistic Gel and 30% Humimic Gel, to closely mimic the Shore hardness of human tissue. Organs were created using Clear Ballistic gel and then coated with talcum powder to make the organs more distinguishable during imaging. A 3D-printed Polycarbonate (PC) skeleton was used to withstand the temperature of the melted gel. Organ hemorrhages were developed by introducing latex balloons filled with water at 3 anatomically correct positions (Al-Zogbi et al., 2021). This study was able to develop a cost effective, anatomically correct FAST phantom. However, ultrasound images of the scan points were not shown, so it is challenging to gauge the anatomical relevance of the model. Also, similar to the commercial trainers, this phantom lacks the ability to perform PTX scanning.

Our research team has recently developed a tissue phantom focused specifically on the PTX injury. In a previous study, we developed a synthetic PTX model using a 3D-printed rib mold combined with ballistic gel. For negative PTX conditions, healthy breathing was simulated by sliding an aerated lung phantom against the inner surface of the rib model. Positive PTX was simulated by imaging the rib model with an air gap between it and the lung model. Quality of the images was validated by training a classifier algorithm on phantom images which resulted in more than 90% accuracy on blind PTX positive and negative images captured in euthanized swine tissue (Boice et al., 2022a). However, this phantom lacked the complete anatomical rib structure needed for integration into an eFAST phantom.

In this effort, we integrate AH, HTX, and PTX positive and negative injury states into a modular tissue phantom as an improved platform for developing and evaluating automation techniques for the eFAST exam. The key objectives for this paper are as follows:

• Integration of positive and negative PTX injury states into a tissue phantom
• Development of RUQ, LUQ and Pelvic views into the same tissue phantom for positive and negative HTX and AH viewpoints.
• Highlight proof of concept deep learning model training with the eFAST phantom for automating image interpretation.

## 2 Materials and methods

### 2.1 Making the phantom

#### 2.1.1 Phantom components

A full torso phantom was developed and composed of the main internal organs that are ultrasonically viewed when performing an eFAST exam. An open access repository containing ready-to-print 3D datafiles was used to obtain models for: the bottom lobe of both lungs, liver, spleen, stomach, both kidneys, bladder, rectum, ribs, costal cartilage, and sternum (Mitsuhashi et al., 2009). Supplementary Table S1 summarizes the material and 3D-printing method used for each part. Fused deposition modelling printing was performed using a Raise3D Pro2 Plus printer (Raise3D, Irvine, CA, United States), and stereolithographic printing was done using FormLabs’ Form2 or Form3L printers (FormLabs, Somerville, MA, United States).

**FIGURE 2:**
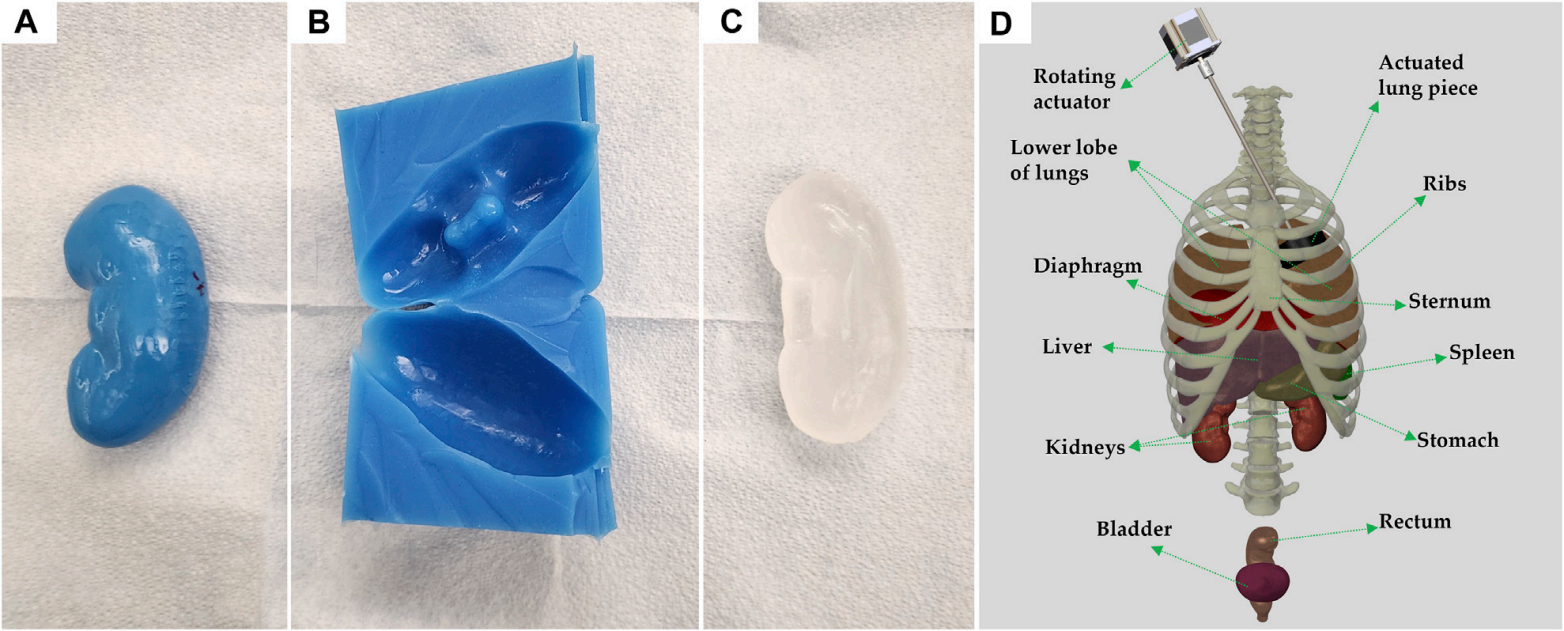
Process of individual organ casting and three-dimensional reconstruction of assembled body parts. **(A)** 3D printed left kidney in polylactic acid. **(B)** Cast of the kidney made of Dragon Skin 10NV. **(C)** CBG organ after removal from cast. **(D)** 3D rendering of all body parts used, in their approximate locations; generated using Solidworks computer aided design (Waltham, MA, United States). Each anatomical component is identified, and the vertebral column is shown for rendering purposes only.

Since the 3D-printed plastic organs have relatively low melting points, a multi-step casting process was used. Each organ was first 3D-printed, and then cast with Dragon Skin 10 NV (Figures 2A, B) (Smooth-On, Macungie, PA, United States). The Dragon Skin’s heat resistant properties made it ideal for use with clear ballistic gel (CBG) (Clear Ballistics, Greenville, SC, United States). Sufficient volumes (varied by organ) of CBG were melted at 130°C and mixed with talc powder (Fasco Epoxies, Ft. Pierce, FL, United States) at 0.25% (w/v) concentration to pour each organ mold (Figure 2C). Specifically for the stomach, lungs, and rectum models, this same CBG mixture was vigorously stirred before pouring to mix in and distribute small bubbles which mimic the natural state of these air-filled organs. Conversely, two different bladder sizes were poured with pure CBG without talc powder, as it is a fluid-filled organ.

Once the ribs, cartilage, and sternum were 3D-printed they were assembled using custom brackets. A schematic of the full assembly, including organs is shown on Figure 2D. In place of the backbone, silicone tubing with notches was used to support the posterior ends of the ribs. To protect the inside of the thoracic cavity, rubber sheets were sown together and attached to the ribs with wires, preventing CBG from leaking into the cavity when pouring the bulk of the torso.

#### 2.1.2 Casting and pouring the full torso

A torso cast was made with EpoxACast HT670 (Smooth On, Macungie, PA, United States) using a plastic mannequin (Amazon, Seattle, WA, United States). The mannequin was modeled after a human male, from the upper thigh region to just above the clavicle and placed supine into the EpoxACast HT670 resin mixture. A rim was fixed normal to the coronal plane and attached around the perimeter of the mannequin allowing the front contour shape to be captured while adding depth to the cast as opposed to fully submerging the mannequin model. This permitted space to position the internal components within the final phantom mold. The bulk of the torso was made of the same CBG with 0.25% (w/v) talc powder mixture. The ribs were placed in the empty cast, lined with rubber sheets, and then layers of liquid CBG mixture were poured until the ribs were covered. Layering was used to allow bubbles to escape after each portion was poured, ensuring minimal bubbles were trapped in the final phantom. The bulk of the phantom was allowed to cool and fully solidify overnight.

After the bulk was ready, the area right behind the ribs was carefully carved to remove the rubber sheets and have access to insert the internal organs. All the carving for organ placement was performed from the back to preserve the front and sides of the torso for ultrasound imaging. The cavity inside the ribs was expanded to the waistline, allowing for enough space to fit the stomach, liver, kidneys, lungs, diaphragm, and spleen. To allow superior access to the thoracic cavity, any neck tissue was removed, leaving the thoracic cavity open from the superior and posterior sides of the phantom.

A second cavity, in the pelvic area, was also carved from the back until there was approximately 2 cm to the surface (front of phantom). This cavity was big enough to fit both bladder sizes along with the rectum. Carving for all areas was performed with a scalpel blade, and a hot knife (Modifi3D, Coalville, United Kingdom). While carving the bulk of the phantom, it was left in the original cast for ease of mobility.

### 2.2 Arranging and imaging each scan point

US imaging was performed using three different US systems: Sonosite Edge (Fujifilm Sonosite, Bothell, WA, United States), Sonosite PX (Bothell, WA, United States), and Terason 3200t (Terason, Burlington, MA, United States). For the chest scan points a linear-array probe from each system was used to collect M-mode images. All other views were scanned with curvilinear and phased-array probes to obtain a 30 s B-mode clip of the scan points. Additional details specific to each scan point are described in the sections below. A polyurethane quick set foam resin (McMaster-Carr, Elmhurst, IL, United States) platform was created to the same dimension as the phantom cast to act as a base for the phantom to lay on its back while US imaging.

#### 2.2.1 Pneumothorax view

PTX baseline images were created by rotating a 2 cm wide and 1.1 cm thick foam ring under an intercostal space in the chest of the phantom. The foam piece was fused with a cyanoacrylate adhesive (Loctite, Düsseldorf, Germany) along the perimeter of an acrylic gear with a diameter of 6.25 cm. The gear was then attached to the end of the rod and placed under the intercostal space region of interest. The rotating motion was created by an actuator (Amazon, Seattle, Washington, United States) powered by a 12 V power supply at 12 revolutions per minute. The motion was transferred to inside the phantom through the neck cavity using a hex-rod. The t-rail containing the actuator was angled to allow complete contact between the intercostal space and the foam. Twelve evenly spaced cuts were made to the foam midpoint in order to give the M-mode images more granularity. Prior to imaging, US gel (Aquasonics Enterprises, Gary, IN, United States) was applied to the foam ring. Rotating the device resulted in baseline images as lung motion is mimicked by rotation. PTX positive images were collected by removing the device from view. A total of four M-mode images for each condition, negative and positive PTX, were captured with the three US systems.

#### 2.2.2 Pelvic view

The bladder and rectum CBG models were attached to each other using melted CBG and placed inside the pelvic cavity. A 10% gelatin mixture dissolved in water and evaporated milk, with flour at 0.25% w/v concentration was added around the organs to fill out the space in the cavity. This mixture has been previously used to simulate tissue in US phantoms (Hernandez-Torres et al., 2022). The gelatin mixture was left to solidify at 4°C for approximately 2 h covered in plastic wrap to prevent gelatin dehydration. To create positive AH injuries, a CBG hypoechoic pocket was cast in a custom-made 3D mold and attached between the bladder and rectum. Once gelatin solidified, the phantom was placed supine on the foam base for US imaging.

#### 2.2.3 Right and left upper quadrant views

A wall was created inside the rib cavity to separate areas designated to PTX and HTX models. The wall was created by placing two laser cut (Dremel, Racine, WA, United States) acrylic panels inside the cavity with CBG poured on top to create a fluid seal. For AH and HTX models, a CBG diaphragm model was developed by spreading melted CBG mixed with 0.25% (w/v) flour into a thin layer. The diaphragm was then attached to the lungs and to the ribs in the thoracic cavity of the phantom using melted CBG. The liver, spleen, stomach, and kidneys were attached to each other using melted CBG as pasting material in their approximate anatomical locations. The CBG organs were then placed inside the rib cavity. A total of 3L of the 10% gelatin mixture simulating tissue was used to fill the cavity space around the organs. The phantom was held at 4°C to allow the gelatin mixture to solidify. To create a positive HTX injury, a sheet of CBG without any air bubbles was placed in between the ribs and lungs. For positive AH injuries, the hypoechoic pocket used for the pelvic view was also attached in between the liver and kidney or spleen and kidney for RUQ and LUQ AH, respectively. Imaging was conducted with the phantom remaining prone in its cast to maintain structural integrity.

### 2.3 eFAST commercial simulator

A commercial eFAST trainer (Simulab, Seattle, WA, United States) was used for scan point view location confirmation and US image comparison. The trainer included two normal patients and three patients with different injury combinations. As the probe goes near the different scan points the software shows representative images or videos of the region. Some of the abdominal scan points offer longitudinal and transverse views, as well as videos. The lung scan points have both B-mode and M-mode images. US scans from the software were screen-recorded and framed, using the procedure described in the next section and shown as representative human images throughout the result sections, for comparison.

### 2.4 Ultrasound image classification algorithm training

#### 2.4.1 Preprocessing data

All ultrasound images and clips captured were named according to the US system they were recorded with, injury type, injury severity, and probe used to scan. For the PTX view, images were split into baseline and positive folders and then processed using MATLAB R2022b (MathWorks, Natick, MA, United States). The images were cropped to remove the user interface of each US system, leaving only the M-mode region of interest. For cropping, pixel coordinates of the upper corner were identified, as well as the length and width of the window of interest. To boost the number of US images, a 4-pixel rolling window was used for further cropping, yielding 108 image segments per M-mode image, following the process described previously (Boice et al., 2022a). The image segments were then resized to 512 × 512 × 3 using MATLAB R2022b batch image processing.

For all the other scan points, frames were extracted from each 30 s clips using ffmpeg via a Ruby script. Images for the pelvic view were separated into baseline and AH positive folders, for binary classification. Images for the RUQ and LUQ were split into three categories: baseline, AH positive, and HTX positive. At this point images were processed with MATLAB to crop and remove the patient information and user interface of each ultrasound system. Similar approaches were used to determine the top left corner, width, and height pixel coordinates used for cropping. The images were then resized to 512 × 512 × 3.

#### 2.4.2 AI model training

A previously developed deep learning architecture, ShrapML, was tuned for image classification of US images (Snider et al., 2022). Briefly, the architecture is an original, Bayesian optimized, convolutional neural network with 6 convolutional and two fully connected layers which uses a RMSprop optimization function with 430k trainable parameters. This architecture has been used to develop a PTX detection model (Boice et al., 2022a), which was used to test the images obtained from the full torso tissue phantom for the PTX scan points. The same algorithm architecture was used to train new models for the other three scan points. Prior to training, images were augmented using X-Reflection, Y-Reflection, X-Translation (−60 to 60 pixels), Y-Translation (−60 to 60 pixels), and Image Rotation (−180 to 180°). Example images after augmentation are shown in Supplementary Figure S1.

Training of the eFAST models was conducted using MATLAB R2022b. Phantom images were processed according to the injury type and were split into 70%, 10%, and 20% for training, validation, and testing, respectively. The models were trained for up to 100 epochs with a validation patience of 5, learning rate of 0.001, and a batch size of 32. Training was performed using either an Asus ROG Strix running Windows 11, 12th gen 14 core i9-12900H (2500 MHz), 16GB RAM, and a NVIDIA GeForce RTX 3070 Ti (8 GB VRAM) or a Lenovo Legion 7 running Windows 11, AMD Ryzen 9 5900HX (3300 MHz), 32GB RAM, and a NVIDIA GeForce RTX 3080 (16 GB VRAM).

#### 2.4.3 AI model performance evaluation

Performance of AI models was evaluated on blind, holdout testing images not used during training. Confusion matrices were created to classify testing performance as true positive (TP, correct injury identification), true negative (TN, correct baseline identification), false positive (FP, injury identified when not present) or false negative (FN, injury not identified when present) depending on the accuracy of the prediction to the ground-truth label. These labels were used to calculate accuracy, precision, recall, specificity, and F1 score using widely accepted calculation approaches (Shri Varsheni, 2021), and to create confusion matrices for each model using GraphPad Prism (San Diego, California). In addition, area under the receiver operating characteristic curve (AUROC) was calculated for each label. For the PTX view, eight previously developed replicate AI models were used to make blind test predictions. Performance metrics were calculated on blind test images for three trained models for RUQ, LUQ, and pelvic AI models, and averages and standard deviations were calculated for each metric. Images from three US machines were merged and treated as a single training set unless otherwise specified.

In addition to performance metrics, Gradient-weighted Class Activation Mapping (Grad-CAM) was used to assess what features in an US image was driving the AI model prediction (Selvaraju et al., 2017). Grad-CAM heat map overlays were created using MATLAB R2022b for a subset of test images. “Hot spots” in the overlay correspond to high weighted regions, driving the model prediction for the respective US image.

## 3 Results

### 3.1 Lung view for pneumothorax detection

**FIGURE 3:**
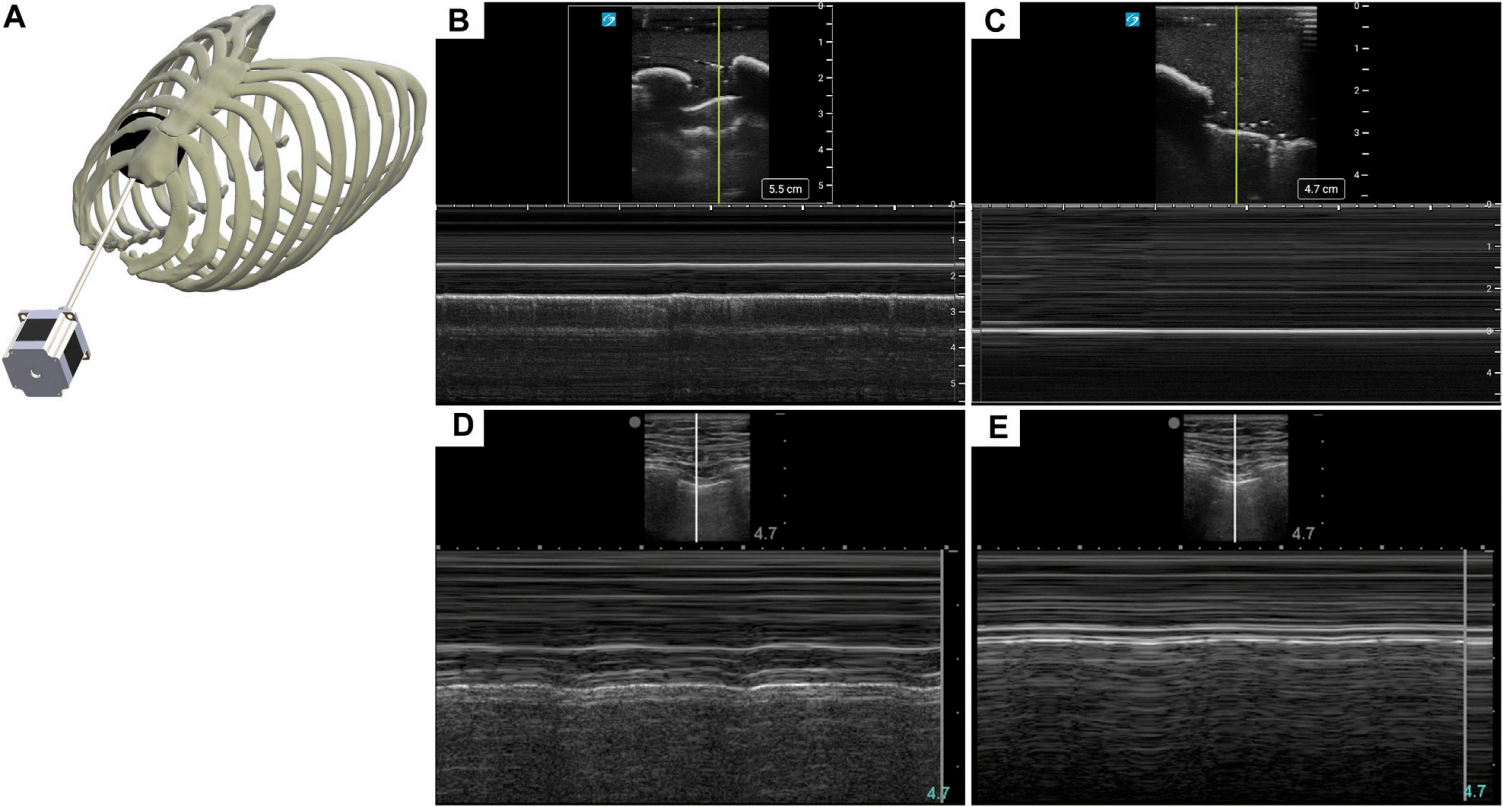
Recreating a pneumothorax injury in the phantom. **(A)** Diagram of the mechanism used to simulate a breathing lung in US for the tissue phantom. **(B)** Baseline or normal (“seashore” sign) M-mode US images collected with the breathing lung mechanism in the phantom. **(C)** PTX positive (“barcode” sign) M-mode US images acquired with the tissue phantom. Representative US images shown in the figure were captured by Sonosite PX. Commercial eFAST simulator human US scans for **(D)** baseline and **(E)** PTX injury.

Diagnosis of PTX using US has key landmarks in both B-mode and M-mode US imaging. While US scanning the full torso phantom with the actuator running (Figure 3A), B-lines and sliding lung were present in real-time, characteristic of healthy breathing lungs. When scanning in M-mode, the “seashore” sign became apparent (Figure 3B) while the actuator was running and then converted to a “barcode” sign (Figure 3C), when the simulated lung was no longer in contact with the pleura. The same patterns can be observed in the commercial eFAST trainer (Figures 3D, E).

**FIGURE 4:**
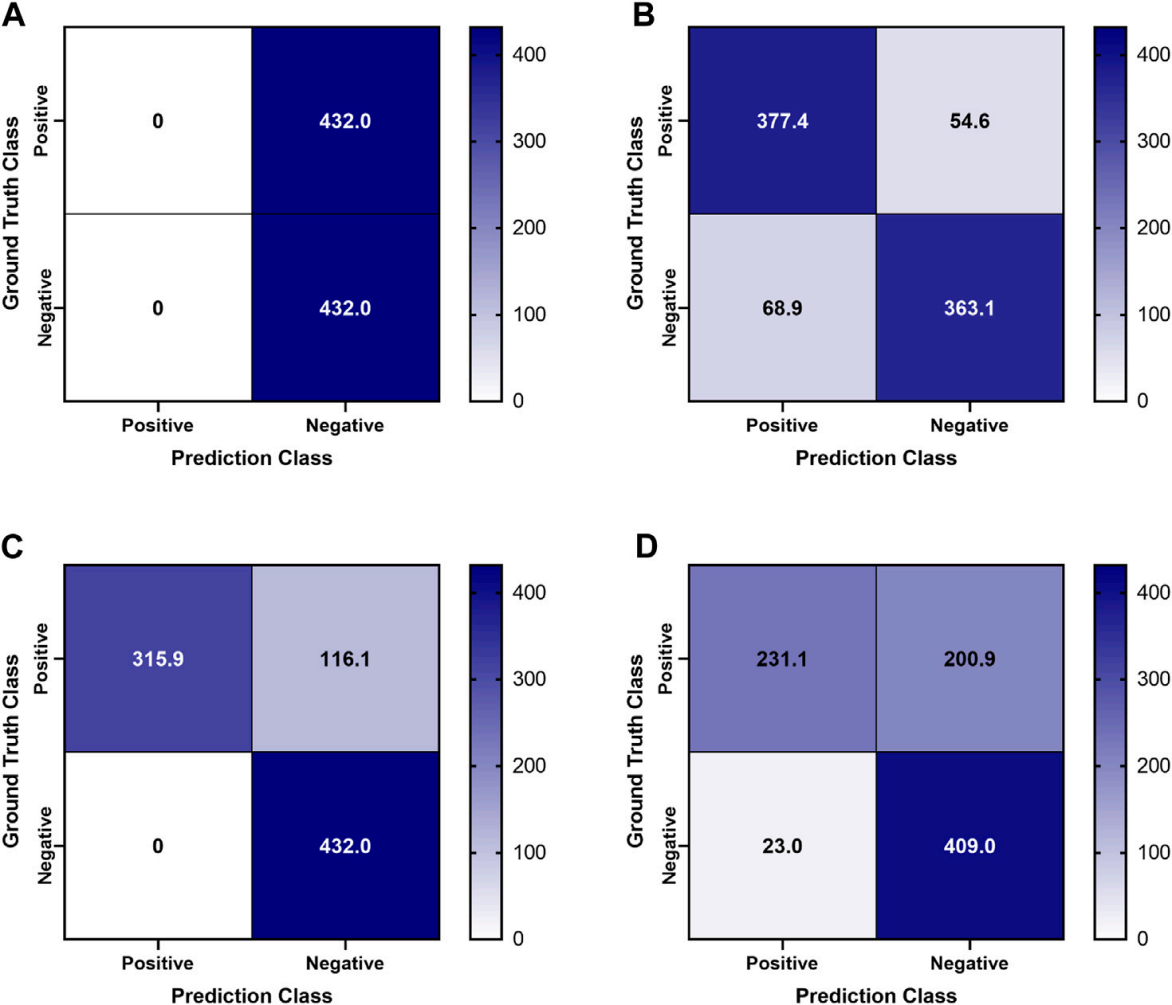
Confusion Matrices for pneumothorax model predictions on test M-mode images collected in the tissue phantom. Previously trained PTX models (*n* = 8) were used to make blind predictions. Average confusion matrix results are shown for US images collected using **(A)** Sonosite Edge, **(B)** Sonosite PX, **(C)** Terason 3200t, and **(D)** average results across all three US systems. Values represent the number of images classified in each confusion matrix category (*n* = 432 PTX positive and *n* = 432 PTX negative images for each US system).

Previously trained AI models, successful at detecting PTX in swine and simple tissue phantom M-mode images, were used to make predictions for the image sets collected in the full torso phantom (Boice et al., 2022a). Initial test results had unsatisfactory performance, leading to the separation of data for each US imaging system to provide a more granular understanding of the results. The Sonosite Edge resulted in all the predictive PTX models (*n* = 8) classifying the images as negative for PTX, resulting in 50% accuracy (Figure 4A). However, this trend did not continue for the other US machines, with Sonosite PX having similar rates for both false positive and false negative outcomes and Terason being biased toward false negative results (Figures 4B, C). The accuracy for both systems was 85%–87% (Table 1). On average, across all the US systems, results were heavily skewed towards a false negative outcome (Figure 4D) due to the Sonosite Edge predictions and had an overall accuracy of 74%.

**TABLE 1:** Performance metrics for pneumothorax model prediction on test M-mode images collected using the developed tissue phantom.

PTX results	Sonosite Edge	Sonosite PX	Terason 3200t	Average
	Average ± StDev	Average ± StDev	Average ± StDev	Average ± StDev
Precision		0.887 ± 0.183	1.000 ± 0.000	0.944 ± 0.080
Recall	0.000 ± 0.000	0.874 ± 0.228	0.731 ± 0.166	0.535 ± 0.469
F1		0.858 ± 0.201	0.834 ± 0.124	0.846 ± 0.017
Accuracy	0.500 ± 0.000	0.857 ± 0.209	0.866 ± 0.083	0.741 ± 0.209
Specificity	1.000 ± 0.000	0.841 ± 0.327	1.000 ± 0.000	0.947 ± 0.092

Results are shown for individual US systems and as an average across the three systems. Performance metrics were averaged across *n* = 8 previously trained pneumothorax models and standard deviations for these replicates were calculated.

**FIGURE 5:**
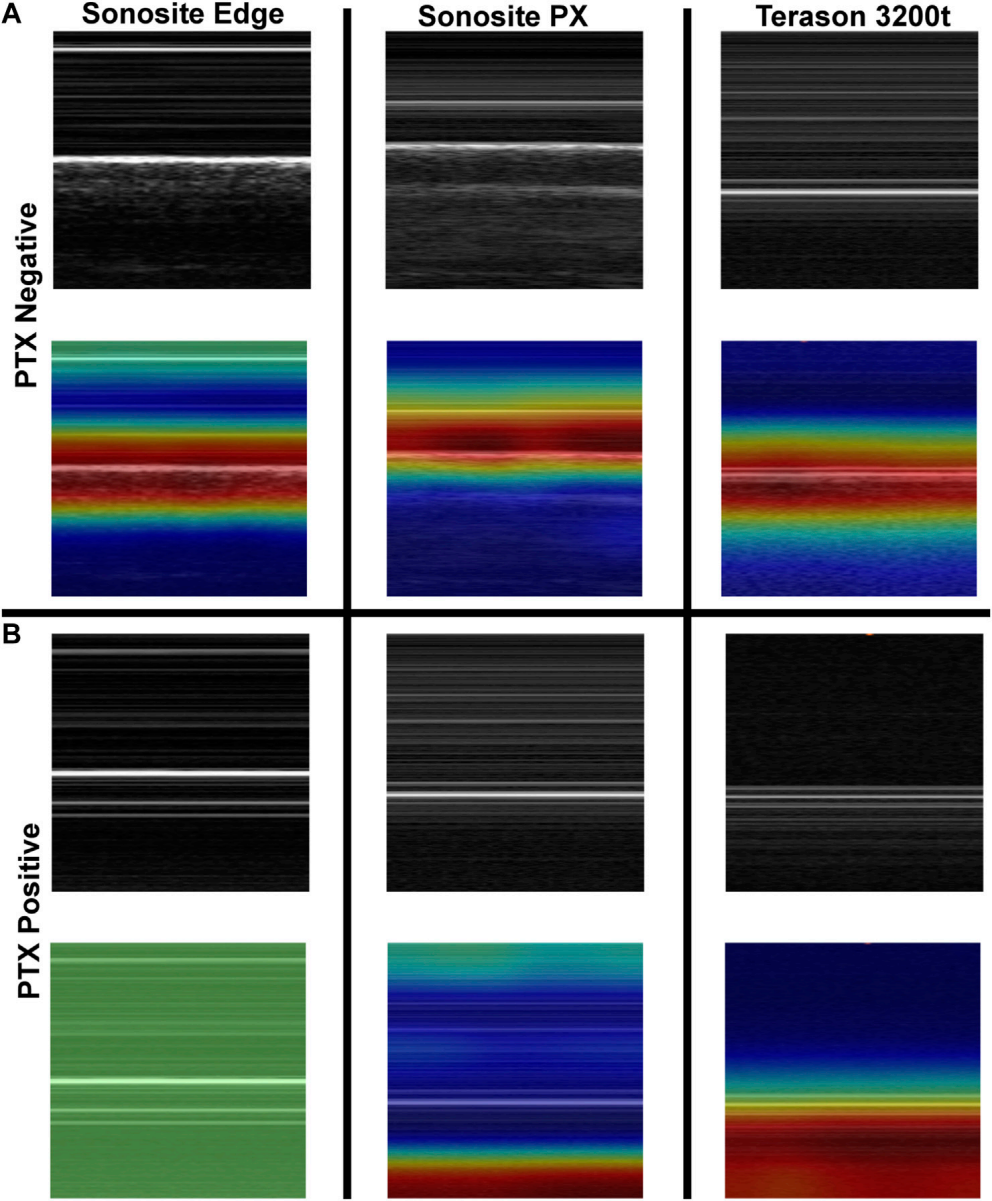
Grad-CAM overlays for pneumothorax AI model predictions on test images collected using the developed tissue phantom. Representative US M-mode scan segments for each US system are shown without and with the Grad-CAM overlay for **(A)** PTX negative and **(B)** PTX positive images. Areas of high importance to the AI model as determined by Grad-CAM are indicated as red-yellow while lower importance regions are denoted as green-blue hues.

To further evaluate differences in predictions, Grad-CAM overlay masks were generated for test images from each US system. Similar, features were being tracked in negative or baseline images for all US systems, with the heat map being focused on the “seashore” sign in the M-mode image (Figure 5). For PTX positive images, all the Sonosite Edge images failed to detect or track any feature, while the majority of predictions from Terason 3200t and Sonosite PX tracked the “barcode” sign on the M-mode image segments (Figure 5). This example highlights the variability that US systems can have on AI predictions and the relevance of including these effects in this stage of model development.

### 3.2 Pelvic view for abdominal hemorrhage

**FIGURE 6:**
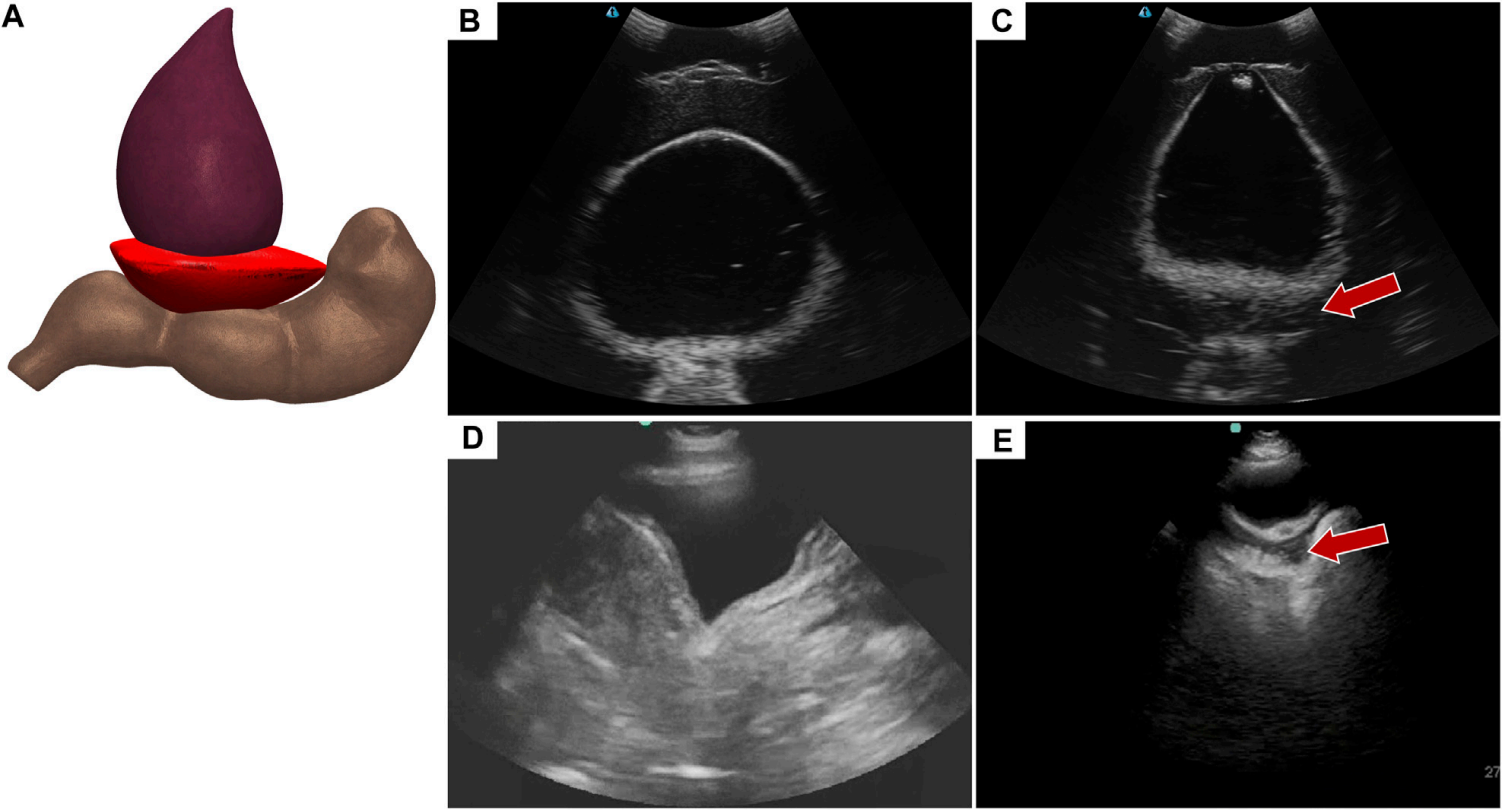
Pelvic view in the eFAST phantom. **(A)** 3D representation of the organ arrangement for the pelvic view with hemorrhage. **(B)** Baseline image showing the bladder and rectum. **(C)** Hemorrhage positive image with blood, represented by hypoechoic pocket, between the bladder and rectum. Hemorrhage region indicated by arrow. Representative US images shown from the phantom were captured with Terason 3200t. Commercial eFAST simulator US scans for **(D)** baseline and **(E)** AH positive.

Abdominal hemorrhage diagnosed in the pelvic view looks at blood pooling behind the bladder towards the rectum (Figure 6A). In the tissue phantom, this was replicated by placing a hypoechoic pocket between the bladder and rectum (Figure 6C). The pelvic view in the full torso phantom for both baseline and AH (Figures 6B, C), resembles the US images from the commercial trainer for the same views and injury (Figures 6D, E), except for US image depth and contrast.

**FIGURE 7:**
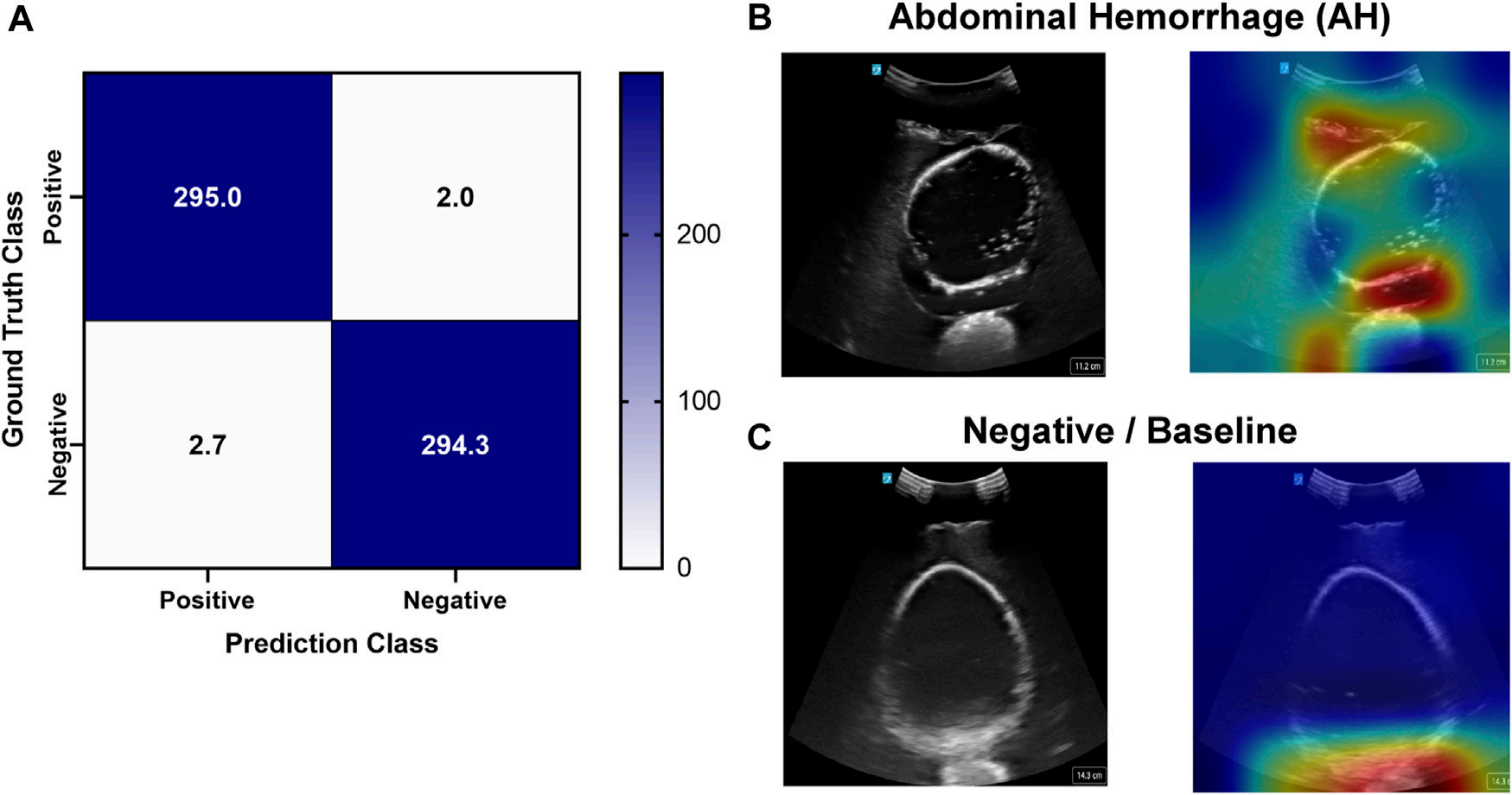
Performance results for an AI classification model trained for the pelvic eFAST view. **(A)** Confusion matrix results for test images grouped into two categories: AH positive and negative for injury. Average number of images per confusion matrix category shown (*n* = 3 trained models, *n* = 297 images per classification category). **(B–C)** AI model Grad-CAM overlays for representative US images for **(B)** AH positive and **(C)** negative predictions. US images are shown without and with heat map overlay. Areas of high importance to the AI model as determined by Grad-CAM are indicated as red-yellow while lower importance spots are denoted as green-blue hues.

Deep learning predictive models for the pelvic view were trained using tissue phantom images as AI models had not been previously developed for this imaging application. A subset of images was used for training (70%) and validation (10%) and included images from three US systems to help overcome prediction biases. Using blind test images (20%) from all US systems, the resulting trained AI model successfully identified true positive and negative images with a low false positive and negative rate (Figure 7A). Evaluating Grad-CAM predictive overlays, the AI was tracking the region below or around the bladder for positive and negative AH predictions which correlates with where fluid would be found (Figures 7B, C). Triplicate deep learning models were trained with a similar accuracy, 99% on average (Table 2).

**TABLE 2:** Summary of performance metrics for the pelvic AI model.

Pelvic results	Average ± StDev
Precision	0.991 ± 0.002
Recall	0.993 ± 0.003
F1	0.992 ± 0.002
Accuracy	0.992 ± 0.002
Specificity	0.991 ± 0.002
AUC^a^	0.998 ± 0.001

Average results and standard deviations are shown for *n* = 3 trained models.

^a^ Area Under the ROC (receiver operating characteristic) Curve.

### 3.3 Right upper quadrant view

**FIGURE 8:**
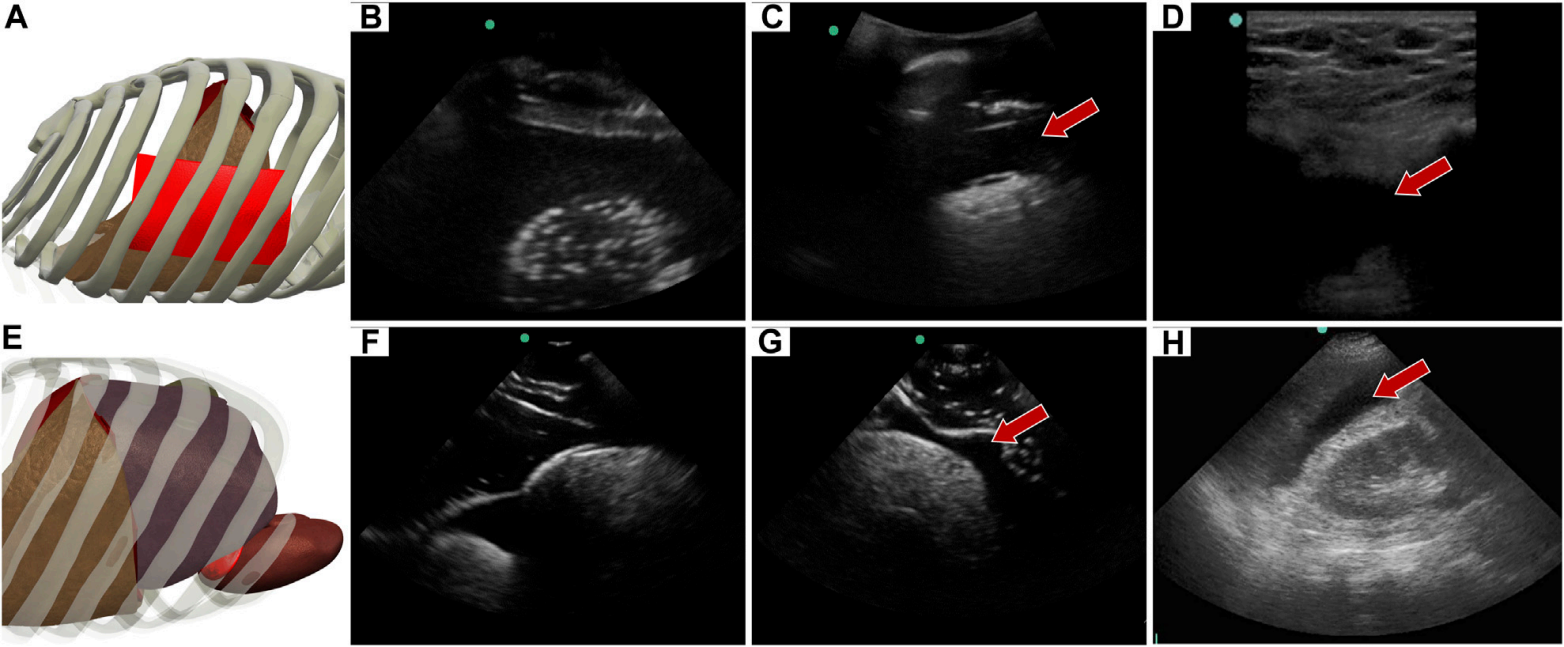
Right upper quadrant view in the phantom. **(A)** 3D representation of the organ arrangement for the RUQ view, focused on HTX. **(B)** Baseline image showing the diaphragm through an intercostal space. **(C)** HTX positive image, blood is indicated by arrow between the pleural spaces. **(D)** Commercial phantom image of positive HTX. **(E)** 3D representation of the organ arrangement for the RUQ view, focused on AH. **(F)** Baseline image of the liver and kidney. **(G)** AH positive image, blood indicated by red arrow separating the kidney and liver. **(H)** Human US image obtained from commercial eFAST trainer. Representative phantom US images shown were captured with Sonosite Edge system.

The abdominal scan point on the right side of the abdomen can look for HTX or AH. When the US image is shallower and focused on the posterior, lower rib area HTX can be diagnosed by blood accumulating within the pleural space. In the developed eFAST phantom, this was simulated by placing a completely clear sheet of ballistic gel (Figure 8A) between the ribs and bubbly lung, generating a HTX positive image in our phantom (Figures 8B, C). US scan focused on the liver and kidney looks at the region known as Morrison’s pouch for any signs of abnormality. Here, differences are noticed if blood is pooling between the liver and kidney (Figure 8E), as placed in the tissue phantom (Figures 8F, G). Images were similar to when comparing to the same anatomical location in the commercial eFAST trainer human images (Figures 8D, H).

**FIGURE 9:**
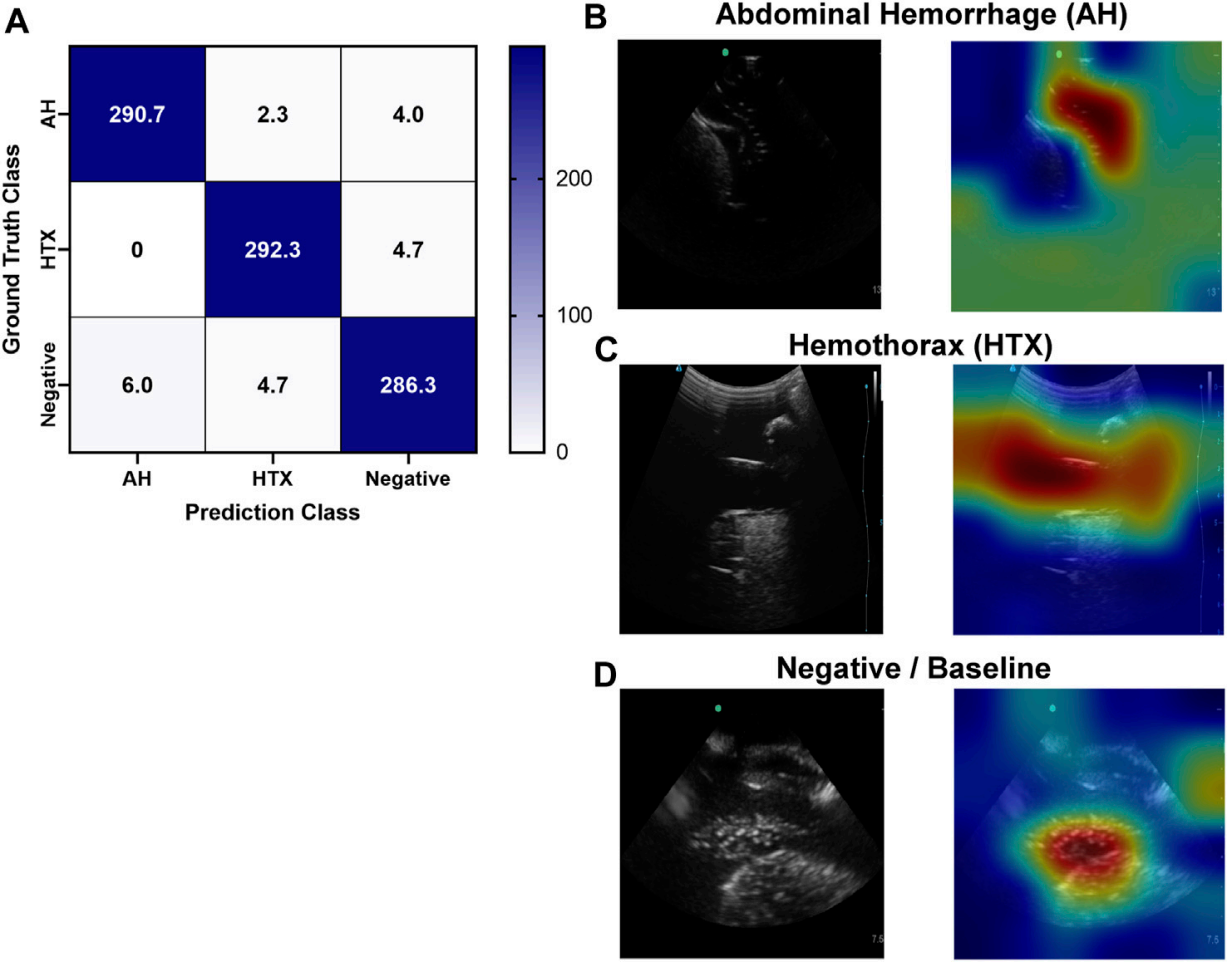
Performance results for an AI classification model trained for the right upper quadrant eFAST view. **(A)** Confusion matrix results for test images grouped into three categories: AH, HTX, and negative for both injuries. Average number of images per confusion matrix category shown (*n* = 3 trained models, *n* = 297 images per classification category) **(B–D)** AI model Grad-CAM overlays for representative US images for **(B)** AH, **(C)** HTX, and **(D)** negative predictions. US images are shown without and with heat map overlay. Areas of high importance to the AI model as determined by Grad-CAM are indicated as red-yellow while lower important spots are denoted as green-blue hues.

Similar to the pelvic view, an AI model was trained for this specific application using the previously developed ShrapML framework. However, the model was modified to allow for three categorical outcomes - AH, HTX, and negative for either injury. A model was successfully trained using US images from all three US systems, with blind image predictions identifying each of three categories with high accuracy and without an obvious bias toward any false category (Figure 9A). Evaluating Grad-CAM overlays, AH-positive predictions were often focused on the area around the hemorrhage region (Figure 9B) and HTX-positive predictions were focused on the middle of the US scan where the thorax hemorrhage effects were most obvious (Figure 9C). Negative predictions had less of a consistent focus, with most heat map attention on the organs at the center of the US scan (Figure 9D). Overall testing accuracies for each of the three categories were 98.6%, 98.7%, and 97.6% for AH, HTX, and negative, respectively (Table 3).

**TABLE 3:** Summary of performance metrics for right upper quadrant AI model.

RUQ Results	Abdominal Hemorrhage	Hemothorax	Negative
	Average ± StDev	Average ± StDev	Average ± StDev
Precision	0.980 ± 0.015	0.977 ± 0.009	0.971 ± 0.006
Recall	0.979 ± 0.015	0.984 ± 0.008	0.964 ± 0.019
F1	0.979 ± 0.014	0.980 ± 0.006	0.967 ± 0.012
Accuracy	0.986 ± 0.009	0.987 ± 0.004	0.978 ± 0.008
Specificity	0.990 ± 0.008	0.988 ± 0.005	0.990 ± 0.008
AUC^a^	0.999 ± 0.001	0.999 ± 0.001	0.998 ± 0.001

Average results and standard deviations are shown for *n* = 3 trained models for each classification category: abdominal hemorrhage positive, hemothorax positive, and negative for both injuries.

^a^ Area Under the ROC (receiver operating characteristic) Curve.

### 3.4 Left upper quadrant view

**FIGURE 10:**
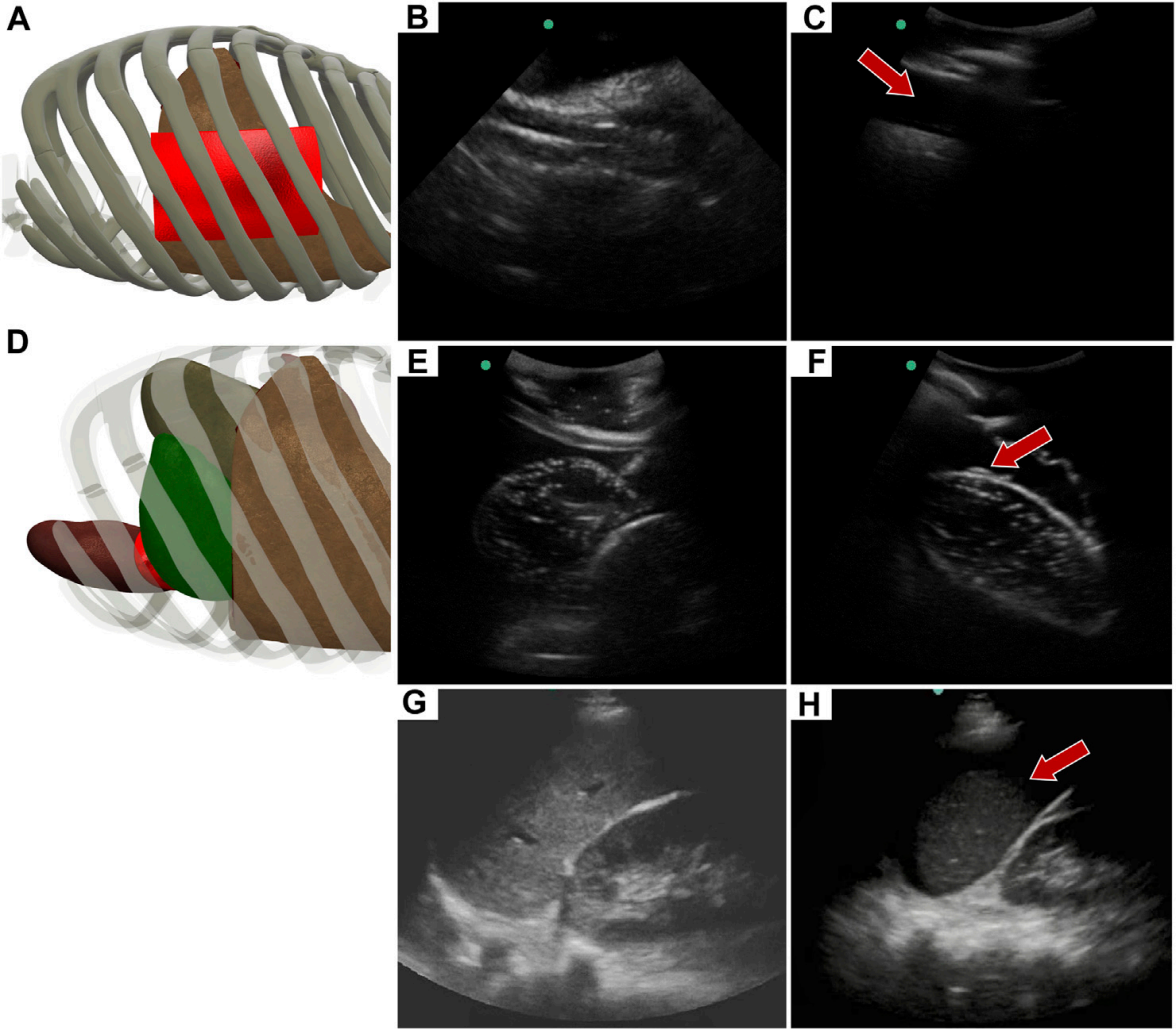
Left upper quadrant view in the phantom. **(A)** 3D representation of the organ arrangement for the LUQ view, focused on HTX. **(B)** Baseline image showing the diaphragm through an intercostal space. **(C)** HTX positive image, blood is indicated by arrow between the pleural space and the lung. **(D)** 3D representation of the organ arrangement for the LUQ view, focused on AH. **(E)** Baseline image of the spleen and kidney. **(F)** AH positive image, blood indicated by red arrow separating the kidney and spleen. Commercial eFAST trainer LUQ US scans for a **(G)** baseline image and an **(H)** AH positive image. Representative phantom US images shown in the figure were captured with Sonosite Edge system.

Similar to the RUQ view, the LUQ scan focuses on the upper left side of the abdomen, where HTX and AH can be diagnosed as well. For HTX, the US diagnosis focuses on fluid accumulating between the pleural spaces (Figure 10A) and shows as a dark fluid band superficial to the lung, visible through an intercostal space (Figures 10B, C) in the full torso tissue phantom. The AH on the left side of the body can be diagnosed by a dark hypoechoic strip of blood between the kidney and spleen (Figures 10E, F), as arranged in the US tissue phantom (Figure 10D). When compared to human US scans (Figures 10G, H), images captured with the tissue phantom were anatomically similar.

**FIGURE 11:**
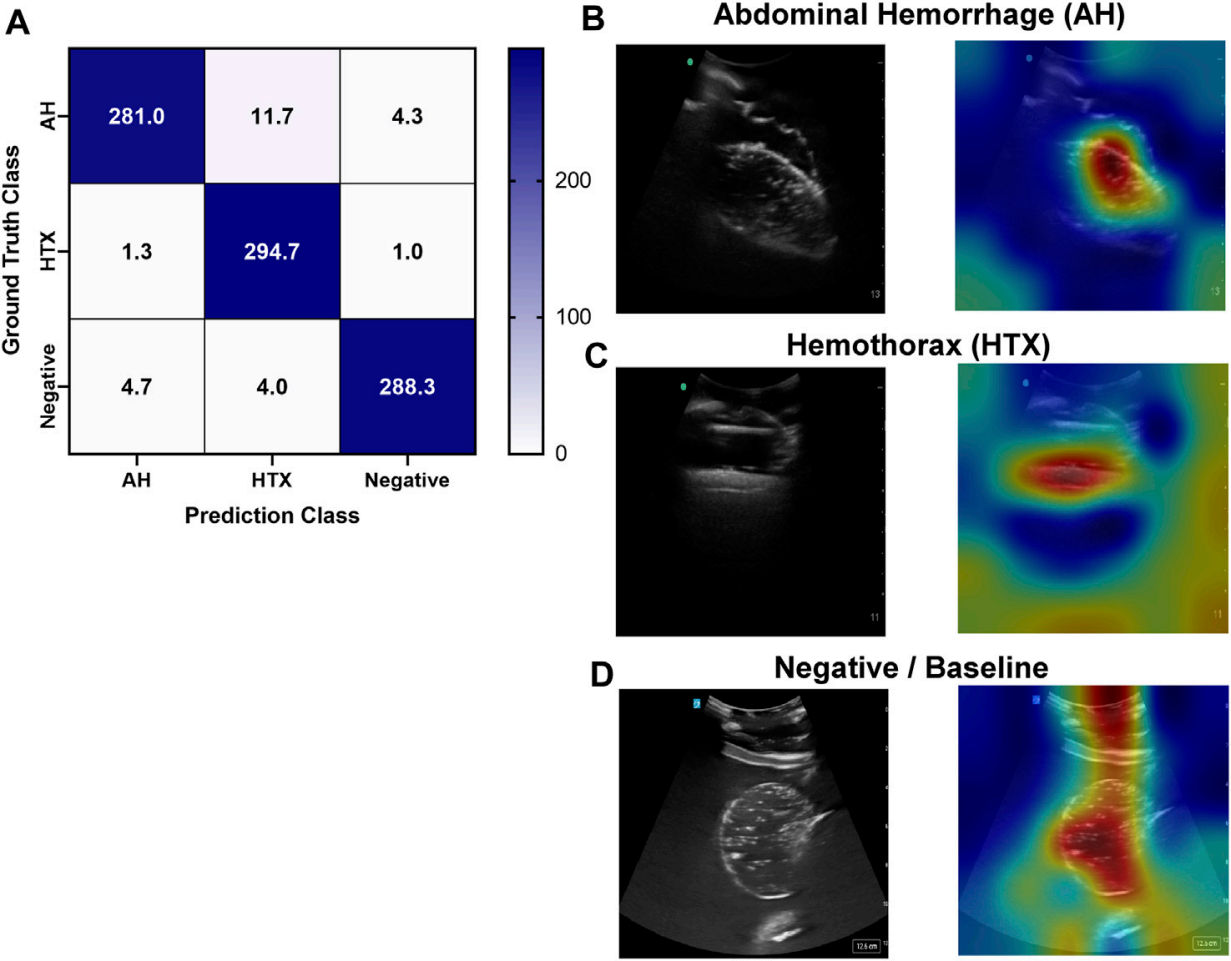
Performance results for an AI classification model trained for the left upper quadrant eFAST view. **(A)** Confusion matrix results for test images grouped into three categories: AH, HTX, and negative for both injuries. Average number of images per confusion matrix category shown (n = 3 trained models, n = 297 image per classification category) **(B–D)** AI model Grad-CAM overlays for representative US images for **(B)** AH, **(C)** HTX, and **(D)** negative predictions. US images are shown without and with heat map overlay. Areas of high importance to the AI model as determined by Grad-CAM are indicated as red-yellow while lower importance spots are denoted as green-blue hues.

An approach similar to the RUQ view was taken to train a three category—AH positive, HTX positive, and negative for both injuries—classification model for the LUQ views. Resulting models had a strong affinity towards true positive and true negative predictions across the three categories (Figure 11A). The AH positive predictions were often tracking the image region where blood was present (Figure 11B) and HTX predictions continued this trend of tracking the hemorrhage region but sometimes detecting the darker area of the overall image (Figure 11C). Negative image predictions were mostly focused on a cross-section of the image and the absence of hemorrhage in those regions (Figure 11D). In summary, model accuracy for the LUQ view for the test set by category was 97.5%, 98.0%, and 98.4% for the AH-positive, HTX-positive, and negative categories, respectively (Table 4).

**TABLE 4:** Summary of performance metrics for left upper quadrant AI model.

LUQ Results	Abdominal Hemorrhage	Hemothorax	Negative
	Average ± StDev	Average ± StDev	Average ± StDev
Precision	0.980 ± 0.029	0.951 ± 0.049	0.982 ± 0.015
Recall	0.946 ± 0.058	0.992 ± 0.011	0.971 ± 0.025
F1	0.962 ± 0.033	0.971 ± 0.026	0.976 ± 0.018
Accuracy	0.975 ± 0.021	0.980 ± 0.019	0.984 ± 0.012
Specificity	0.990 ± 0.015	0.973 ± 0.028	0.990 ± 0.015
AUC^a^	0.997 ± 0.003	0.997 ± 0.002	0.998 ± 0.002

Average results and standard deviations are shown for *n* = 3 trained models for each classification category: abdominal hemorrhage positive, hemothorax positive, and negative for both injuries.

^a^ Area Under the ROC (receiver operating characteristic) Curve.

## 4 Discussion

Ultrasound imaging is a critical tool for life-saving triage decisions during emergency and military medicine. However, the skill threshold for US image acquisition and interpretation makes wide use of this technology challenging. Automating US image interpretation has the potential to lower this threshold if properly designed, which requires large image datasets and troubleshooting to evaluate real-time implementation of this technology. Tissue phantoms, if properly developed, can accelerate automation technology development by allowing initial AI model training and troubleshooting to be possible without the need for animal or human testing. The eFAST tissue phantom in this effort helps meet this need by incorporating AH detection scan points with thoracic PTX and HTX detection.

The eFAST phantom successfully integrated injury sites for four out of the five scan areas evaluated during the exam. The PTX methodology built on a simple 2D phantom we previously developed by integrating a similar motion concept to mimic lung motion in a realistic rib cage. Resulting images resemble those from the prior phantom and human US scans. However, the methodology in its current form lacks the capability of altering respiratory rate in baseline images and the creation of lung points which are often looked for when diagnosing PTX clinically. HTX was accurately detected at the right and left side scan points and mimicked human US images, while AH was detectable across three abdominal scan points. HTX and AH can be visualized simultaneously or independently at a scan point to create more variations in the phantom setup when simulating an eFAST exam. The bladder volume present at the pelvic scan point was modular to represent a more and less full bladder as the effect of that on successfully identifying AH at this scan point is well known (Richards and McGahan, 2017; Rowland-Fisher and Reardon, 2021). The only eFAST scan point not included was the subxiphoid view for cardiac assessment. Creating an US phantom analogue that mimicked heart motion and had realistic heart chamber structure for proper eFAST examination was not possible with the setups needed at the RUQ and LUQ viewpoints and PTX scan sites. Tissue phantoms exist for the cardiac view and even hemopericardium detection which could be used in conjunction with this tissue phantom to allow for inclusion of this eFAST scan point (CAE Healthcare, 2022).

To demonstrate a use case for the eFAST tissue phantom, we evaluated existing or newly trained AI models for the various scan points using images collected in the eFAST protocol. The new models for AH and HTX for three different scan points were successful at accurately detecting these injuries at more than 95% accuracy for all instances. While one of the benefits for the tissue phantom is that these different viewpoints can be reformatted to increase subject variability, it is still less than biological noise, so these high performances were expected. However, efforts were taken during AI model training to prevent overfitting. A widely used approach is to augment image inputs so that AI models less easily focus on image artifacts not associated with the injury. For this effort, we used rotation, flip, zoom, translation augmentations similar to approaches successfully used in other AI US imaging efforts (Hussain et al., 2017; Xu et al., 2022; Snider et al., 2023). Another approach taken was to include validation patience during training so that training ceased if validation loss did not decrease for five training epochs. This prevents model overfitting during 100s of training epochs and was triggered within 20–40 epochs for all training performed. With the limited datasets at this point, the ultimate validation was the Grad-CAM overlays that highlight the region on the image that is driving predictions. With the described overfitting prevention methods, Grad-CAM overlays showed the majority of predictions were tracking proper injury locations. However, some images continue to track image artifacts not associated with injury, so more training data and subject variability will be needed to further improve these developed AI models. Regardless, this use case still highlights the potential use for this phantom model.

Unlike the other injury conditions, we have previously developed a model for PTX detection from segments of M-Mode images which was successful for blind PTX detection in swine images (Boice et al., 2022a). This deep-learning model was only trained on a simple PTX tissue phantom. Using this trained model, prediction performance was heterogeneous based on US imaging system. Specifically, images from one system were 50% accurate, with every prediction failing to see a PTX injury, while the other US systems were more than 85% accurate, achieving slightly lower performance than the swine image predictions at 93% (Boice et al., 2022a). Image acquisition bias on AI model predictions is a known issue that includes medical imaging equipment bias among other potential biases that can limit model generalization (Drukker et al., 2023). The availability of US technology differs, and the technology is always advancing, so it is imperative that instrument noise be accounted for during training to make AI models for eFAST be more robust for implementation on multiple platforms.

There are some limitations with the eFAST tissue phantom and trained AI models that should be noted in this work. First, the eFAST phantom is slow to create compared to using commercially available trainers. The bulk of the tissue phantom housing the organs only needs to be made once, but the gelatin embedded US imaging sites must be newly cast each time which can take approximately 2 h to solidify prior to imaging. Second, while the eFAST phantom contains some subject variability, it pales in comparison to the variability expected to be seen in a human subject population. The re-pouring of the US scan regions can assist with this, but organ sizes and hemorrhage severity were not varied in this work, with the exception of the fullness of the bladder. Third, the PTX negative lung motion can only be viewed at a single rib space; the mechanism also does not allow for lung point generation in which partial PTX positive and negative views are evident at a single intercostal space, a phenomenon clinically used to identify PTX (Skulec et al., 2021). Lastly, the AI models developed using the eFAST phantom need to be validated and transfer-learned with human or animal images before they are suitable for use beyond this tissue phantom platform.

Next steps to expand on the current tissue phantom and its applications will extend in three directions. First, the phantom will be applied for real-time AI and US image acquisition applications to prepare technology for eFAST automation. This includes integration of AI with US hardware and the use of robotics and computer vision algorithms to identify proper scan points and acquire images. Next, the eFAST phantom will be further updated to include an external skin layer with recognizable features, such as nipples, to improve its use with computer vision applications. Other improvements could include more noise in organ size, placement, and injury severity to expand on the robustness of the AI training potential for the tissue phantom. Lastly, the eFAST phantom will be validated against a wider range of eFAST US scans acquired in humans which can be used to further improve on eFAST images or to augment images through generative adversarial networks to create synthetic images that merge phantom and human images to create a better training network for deep learning images (Creswell et al., 2018; Yi et al., 2019).

## 5 Conclusion

Ultrasound imaging can be a critical triage tool not just in hospital settings but in military or more remote medicine if the skill threshold can be lowered. Lowering this skill threshold is critical for prolonged field care medical scenarios where trained personnel may not always be available. With proper training setups such as the eFAST phantom developed in this work, AI image interpretation models and automated methodology evaluation and troubleshooting can be accelerated. We demonstrate this usability by training AI models to automate US image interpretation, and these models were successfully developed for each eFAST scan point with a high accuracy while identifying the proper region of the image. Continuing this research effort into real time AI integration with US devices and pairing with means of automated image acquisition will significantly reduce the skill threshold and allow this critical triage tool to be more widely used.

## Data Availability

The datasets presented in this article are not readily available because they have been collected and maintained in a government-controlled database that is located at the US Army Institute of Surgical Research. As such, these data can be made available through the development of a Cooperative Research & Development Agreement (CRADA) with the corresponding author. Requests to access the datasets should be directed to Eric Snider, eric.j.snider3.civ@health.mil.
