# Supplementary material for: An extended focused assessment with sonography in trauma ultrasound tissue-mimicking phantom for developing automated diagnostic technologies
Source: Front Bioeng Biotechnol. 2023 Nov 14;11:1244616. doi: 10.3389/fbioe.2023.1244616 (PMC10682760; doi:10.3389/fbioe.2023.1244616)
Supplement: Supplementary file 1 [file DataSheet1.docx]

**Supplementary Information**

**Supplementary Table 1. Material for all 3D printed body parts.** Casts were made for all internal organs, the bulk of the phantom was poured around the ribs, cartilage, and sternum.

| **Body Part** | **Material** | **Printer** |
| --- | --- | --- |
| Lungs (bottom lobe) | Polylactic acid | Raise3D Pro2 Plus |
| Liver | Polylactic acid | Raise3D Pro2 Plus |
| Spleen | High Temp Resin | FormLabs Form2 |
| Kidneys | Polylactic acid | Raise3D Pro2 Plus |
| Stomach | Polylactic acid | Raise3D Pro2 Plus |
| Bladder full | High Temp Resin | FormLabs Form2 |
| Bladder semi-full | Tough Resin | FormLabs Form3 |
| Rectum | Polylactic acid | Raise3D Pro2 Plus |
| Ribs | Polycarbonate | Raise3D Pro2 Plus |
| Costal Cartilage | Flexible Resin | FormLabs Form3L |
| Sternum | Polycarbonate | Raise3D Pro2 Plus |


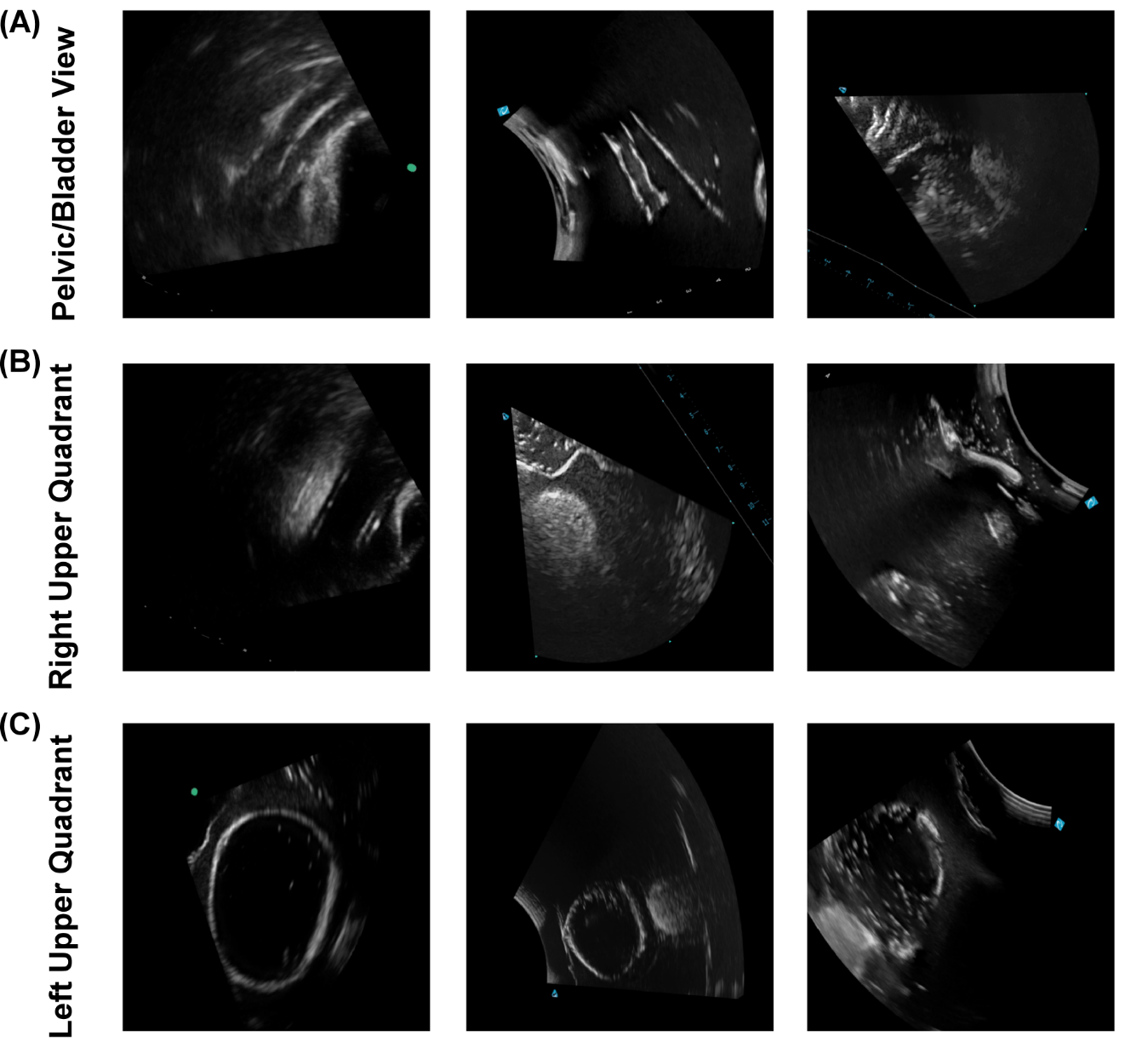


**Supplementary Figure 1**. Example US images after image augmentation has been randomly performed for (A) pelvic view, (B) right upper quadrant view, and (C) left upper quadrant view images.
